# Validating the inhibitory effects of d- and l-serine on the enzyme activity of d-3-phosphoglycerate dehydrogenases that are purified from *Pseudomonas aeruginosa*, *Escherichia coli* and human colon

**DOI:** 10.1186/s13099-019-0315-8

**Published:** 2019-06-30

**Authors:** Jun Okuda, Syouya Nagata, Masashi Yasuda, Chigusa Suezawa

**Affiliations:** Division of Microbiology, Department of Medical Technology, Kagawa Prefectural University of Health Sciences, Kagawa, Japan

**Keywords:** *serA*, d-3-phosphoglycerate dehydrogenase, *P. aeruginosa*, Human colon, d- and l-serine, Bacterial translocation, Gut-derived sepsis

## Abstract

**Background:**

We previously demonstrated that the *serA* gene is associated with bacterial pathogenicity, including bacterial penetration through the Caco-2 cell monolayers, bacterial motility, bacterial adherence, and fly mortality. l-Serine is known to inhibit the d-3-phosphoglycerate dehydrogenase (PGDH) activity of the SerA protein, and it significantly reduced the bacterial pathogenicity as described above. We also demonstrated that in a PGDH assay using crude extracts isolated from overnight cultures of *E*. *coli* overexpressing the *P*. *aeruginosa serA* gene, l-serine inhibited the PGDH activity of the SerA protein. The basal PGDH activity of the negative control strain was high, presumably due to contamination of unknown proteins in the crude extracts. Therefore, to further confirm the direct inhibition of PGDH activity of *P*. *aeruginosa* SerA by l-serine, we purified and characterized the PGDH from *P. aeruginosa* and compared it with the previously characterized PGDHs from *E. coli*, and the human colon as controls.

**Results:**

Optimum pH and ionic strength of the purified PGDHs were different depending on the three species; optimal activity of *P. aeruginosa* PGDH was at pH 7.5 with 50–100 mM Tris–HCl, *E. coli* PGDH was at pH 8.5 with 100–200 mM Tris–HCl, and human PGDH was at pH 9.0 with 100–200 mM Tris–HCl. The addition of l-serine reduced the activity of PGDH from *P. aeruginosa* and *E. coli*, but not the PGDH from human colon. The median inhibitory concentration (IC_50_) of l-serine was 630 μM for *P. aeruginosa* and 250 μM for *E. coli*, while IC_50_ of d-serine was much higher than that of l-serine; 76 mM in *P. aeruginosa* PGDH and 45 mM in *E. coli* PGDH.

**Conclusions:**

These results suggest that l-serine significantly repressed *P. aeruginosa* pathogenicity through direct inhibition of the PGDH activity, but was not able to inhibit the human PGDH activity. Oral administration of l-serine to compromised hosts might interfere with bacterial translocation and prevent gut-derived sepsis caused by *P. aeruginosa* through inhibition of the function of the *serA* gene product.

## Background

*Pseudomonas aeruginosa* is the causative agent of various opportunistic infections, including gut-derived sepsis. We previously demonstrated that the *serA* gene is associated with the bacterial pathogenicity, and has a role in promoting the bacterial penetration through the Caco-2 cell monolayers, which was accompanied by decreased swimming and swarming motility, bacterial adherence, and fly mortality [1]. Further, we previously investigated whether l-serine, which is known to inhibit the d-3-phosphoglycerate dehydrogenase (PGDH) activity of the SerA protein, significantly reduces the known phenotypes associated with bacterial pathogenicity. Consequently, the addition of l-serine was found to significantly reduce the phenotypes associated with the bacterial pathogenicity, including bacterial penetration through Caco-2 cell monolayers, bacterial swimming and swarming motility, bacterial adherence, and fly mortality [1]. Furthermore, we show that in a PGDH assay using crude extracts that were isolated from overnight cultures of *E*. *coli* overexpressing the *P*. *aeruginosa serA* gene, l-serine directly inhibited the PGDH activity of the SerA protein. The background PGDH activity of the negative control strain was high, presumably due to contaminated proteins in the crude extracts. Therefore, to further confirm the direct inhibition of PGDH activity of *P*. *aeruginosa* SerA by l-serine, we purified the PGDH from *P. aeruginosa* using the glutathione S-transferase (GST) fusion protein system which is used for high-level expression and efficient purification of recombinant proteins.

As described in detail previously [2, 3], the *serA* gene encodes the d-3-phosphoglycerate dehydrogenase (PGDH) and catalyzes the first step in serine synthesis by utilizing NAD^+^ as a cofactor in *Escherichia coli*. It is known that the activity of PGDHs derived from certain bacterial species including *E. coli*, can be allosterically inhibited by l-serine, the end product of the serine synthesis pathway, due to a conformational change in the three-dimensional structure of PGDH upon binding of l-serine [2, 3]. On the other hand, the activity of PGDHs isolated from rat and chicken livers appears not to be influenced by the addition of l-serine [4–6]. As described in detail previously [7, 8], this difference in the inhibitory effects of l-serine on the PGDH activity among these species seems to be dependent on the difference in the amino acid sequence and the three-dimensional structure of each PGDH. PGDHs consists of at least three different structural motifs that have been classified as types I, II, and III as described in detail previously [7–10]. As described in detail previously [6–8, 10], the PGDHs from certain bacteria including *E. coli, P. aeruginosa, H. influenza,* and the simple eukaryotes such as yeast, *Leishmania*, and *Neurospora*, retain the type II motif, which contains three distinct domains called the cofactor or nucleotide-binding domain, the substrate-binding domain, and the C-terminal regulatory or the serine binding domain. The C-terminal regulatory or the serine binding domain is also called as the ACT (aspartate kinase-chorismate mutase-tyrA prephenate dehydrogenase) domain and is responsible for l-serine binding and the regulation of PGDH activity; in *E. coli* PGDH, there are critical amino acid residues which are needed for l-serine binding as described in detail previously [7, 8]. Other bacteria, including *Mycobacterium tuberculosis*, *Bacillus subtilis*, and the higher eukaryotes, including mouse, rat, and human, possess the type I motif which harbors a large polypeptide insertion, which is called as ASB (allosteric substrate binding) domain, in the C-terminal fragment which follows the substrate binding domain as described in detail previously [7, 8]. Some organisms, including *Pyrococcus*, *Rhodopseudomonas, Clostridium, Entamoeba histolytica, Bacteroides fragilis*, and *Porphyromonas gingivalis*, have the type III motif which lacks the C-terminal regulatory domain as described in detail previously [7, 8]. Furthermore, there are two forms of the type III motif depending on whether lysine (type K) or histidine (type H) exists at the active site as described in detail previously [7, 8, 10] .

In the present study, we performed the purification and characterization of the PGDH from *P. aeruginosa* PAO1 strain by exploiting the GST fusion protein system as there is no report yet available on the characterization of the *P. aeruginosa* PGDH. Furthermore, we determined the median inhibitory concentration (IC_50_) of d- and l-serine against the purified PGDH isolated from the *P. aeruginosa serA* gene.

## Materials and methods

### Bacterial strains

*Escherichia coli* DH5α strain was purchased from TOYOBO, Japan. *E. coli* BL21 strain was used to express the GST fusion protein and was purchased from GE Healthcare, Japan [11].

### Sequence homology

All sequences used in this study were obtained from the National Center for Biotechnology Information (https://www.ncbi.nlm.nih.gov/). Sequence alignments were performed using the ClustalW (http://clustalw.ddbj.nig.ac.jp/) and the EMBOSS Needle (https://www.ebi.ac.uk/Tools/psa/emboss_needle/).

### Cloning of *P. aeruginosa* serA gene

To construct the plasmid used for the expression of glutathione S-transferase (GST)—*P. aeruginosa* PGDH fusion protein, *Bam*HI–*Xho*I fragment carrying *P*. *aeruginosa serA* ORF was amplified by LA Taq DNA polymerase (TAKARA) under conditions recommended in the manufacturer’s protocol with 5-PA-serA-BamHI-ATG-356477 (5ʹ-GAGAGGATCCATGAGCAAGACCTCTCTCGA-3ʹ) and 3-PA-serA-Xho1-end-355248 primers (5ʹ-GAGACTCGAGTTAGAACAGCACGCGGCTAC-3ʹ); this insert corresponds to the nucleotides 355248 to 356477 in the PAO1 genome sequence (https://www.pseudomonas.com). The *Bam*HI–*Xho*I fragment was ligated into the *Bam*HI–*Xho*I site of pGEX-6P-1 (GE Healthcare) [12], and the resultant plasmid (pGEX-6P-1-P*serA*) was transformed into *E*. *coli* DH5α. The plasmid was isolated from *E. coli* DH5α and was transformed into *E. coli* BL21. The resultant transformant was designated as BL21 (pGEX-6P-1-P*serA*).

### Cloning of *E. coli* serA gene

To construct the plasmid used for the expression of the GST-*E. coli* PGDH fusion protein, *Sma*I-*Xho*I fragment carrying the *E. coli serA* ORF was amplified by LA Taq DNA polymerase (TAKARA) under conditions recommended in the manufacturer’s protocol with 5-ECW-serA-Sma1-ATG-2966687 (5ʹ-GAGACCCGGGTATGGCAAAGGTATCGCTGG3ʹ) and 3-ECW-serA-Xho1-end-2965455 (5ʹ-GAGACTCGAGTTAGTACAGCAGACGGGCGC-3ʹ) primers; this insert corresponds to the nucleotides 2965455 to 2966687 in the *Escherichia coli* strain K-12 substrain W3110 substrain ZK126 genome (https://www.ncbi.nlm.nih.gov). The *Sma*I–*Xho*I fragment was ligated into the *Sma*I–*Xho*I site of pGEX-6P-1, and the resultant plasmid (pGEX-6P-1-E*serA*) was transformed into *E*. *coli* DH5α. The plasmid was isolated from *E. coli* DH5α and was transformed into *E. coli* BL21. The resultant transformant was designated as BL21 (pGEX-6P-1-E*serA*).

### Cloning of the human serA gene

To construct the plasmid used for the expression of the GST-human PGDH fusion protein, *Bam*HI–*Xho*I fragment carrying the human *serA* ORF was amplified by LA Taq DNA polymerase (TAKARA) with primers 5-Human-serA-BamH1-ATG (5ʹ-GAGAGGATCCATGGCTTTTGCAAATCTGCG-3ʹ) and 3-Human-serA-Xho1-end (5ʹ-GAGACTCGAGTTAGAAGTGGAACTGGAAGG-3ʹ); this insert corresponds to nucleotides 137–1742 in the *Homo sapiens* phosphoglycerate dehydrogenase mRNA (cDNA clone MGC:18226 IMAGE:4156703) (https://www.ncbi.nlm.nih.gov).

Human Colon Plasmid cDNA library (Stratagene, #982261) was amplified again on solid medium plate, and plasmid DNA was purified from the amplified library with a plasmid extraction kit (Qiagen).

PCR was carried out in a 50 μL reaction mix containing 200 mM dNTPs (each), 315 ng of human colon plasmid DNA, 0.2 mM primers (each), 5 μL of Takara LA PCR buffer, 2.5 mM MgCl_2_, and 5 U of Takara LA *Taq* polymerase. The protocol was performed as follows: 2 min at 94 °C followed by 35 cycles of 94 °C for 30 s, 58.6 °C for 30 s, and 72 °C for 2 min, and an extension step at 72 °C for 8 min. PCR product of amplicon of 1.6 kb was cloned into pGEMeasy T-vector (Promega), the resultant plasmid (pGEM-H*serA*) was transformed into *E*. *coli* DH5α. The plasmid was isolated from *E. coli* DH5α, and then digested with *Bam*HI and *Xho*I and the digested fragment carrying the human *serA* ORF was ligated into the *Bam*HI–*Xho*I site of pGEX-6P-1, and the resultant plasmid (pGEX-6P-1-H*serA*) was transformed into *E*. *coli* DH5α. The plasmid was isolated from *E. coli* DH5α and transformed into *E. coli* BL21. The resultant transformant was designated as BL21 (pGEX-6P-1-H*serA*).

### Purification of the recombinant PGDHs

The recombinant PGDHs were isolated from the BL21 (pGEX-6P-1-P*serA*), BL21 (pGEX-6P-1-E*serA*) or BL21 (pGEX-6P-1-H*serA*) culture as described below. A 20 mL aliquot of BL21 (pGEX-6P-1-P*serA*), BL21 (pGEX-6P-1-E*serA*) or BL21 (pGEX-6P-1-H*serA*) culture grown in LB broth supplemented with 100 μg/mL of ampicillin was inoculated into 200 mL of fresh LB broth supplemented with 100 μg/mL of ampicillin, incubated at 37 °C for 3 h. PGDH expression was then induced by the addition of 2 mM isopropyl-β-d-thiogalactopyranoside and then incubated at 37 °C for another 1 h. The bacterial cells were harvested by centrifugation at 4000×*g* for 5 min, and the bacterial pellet was resuspended in 12 mL of 1X PBS. After the addition of lysozyme (0.91 mg/mL), the cells were disrupted by sonication for 150 s. After the cell debris was removed by centrifugation at 20,000×*g* for 10 min, 400 μL of 50% slurry of GST-Accept (Nakalai Tesque) was added to the supernatant. After mixing with rotation for 1 h, the cell lysate was centrifuged and the GST-bound proteins were collected. The resulting GST-Accept (containing GST-protein) was washed five times with precision buffer (100 mM Tris–HCl pH 7.5, 150 mM NaCl, 1 mM EDTA, 1 mM DTT) and was finally resuspended in 400 μL of the precision buffer. After addition of precision proteinase (30 units/mL), the tube was mixed by rotation for 15 h and the supernatant containing the PGDH was collected by centrifugation at 4000×*g* for 1 min. The purified PGDH was the used for further analysis including the PGDH activity assay. The amount of protein was measured by TaKaRa Bradford Protein Assay Kit (Takara-bio, Japan). The purified PGDH was applied to 12% SDS-PAGE, after which the proteins were stained using Rapid Stain CBB Kit (Nacalai Tesque).

### PGDH activity assay

PGDH activity assay was carried out as described previously [13] with a modification. Briefly, PGDH activity was measured by measuring an increase in absorbance at 339 nm in the presence of NAD (Nakalai Tesque) and 3-phosphoglycerate (3PG; Sigma-Aldrich, USA). The assays were performed in triplicate and contained the reaction mixture with 100 mM Tris–HCl buffer (*P. aeruginosa* PGDH: pH 7.5, *E. coli* PGDH: pH 8.5, human PGDH: pH 9.0) containing 5 mM EDTA (Wako, Japan), 1 mM DTT (Wako), 10 mM hydrazine (Wako) and 2 mg/mL NAD. The reaction was started with 15 mM 3-phosphoglycerate and the formation of NADH was measured spectrophotometrically at 339 nm. The enzyme activity (unit per mg protein) was calculated by following the enzyme unit reported previously [14, 15].

### Inhibition of PGDH by l-serine or d-serine

PGDH activity assay was performed in the presence of the designated concentration of l-serine or d-serine to evaluate 50% inhibitory concentration (IC_50_) of l-serine or d-serine against PGDH. The IC_50_ was determined as the concentration of l-serine or d-serine that reduced the PGDH activity by 50% as compared to the PGDH activity without the inhibitor.

### Statistical analysis

Statistical analysis was performed using a two-tailed *t* test.

## Results

### Sequence homology

**Fig. 1:**
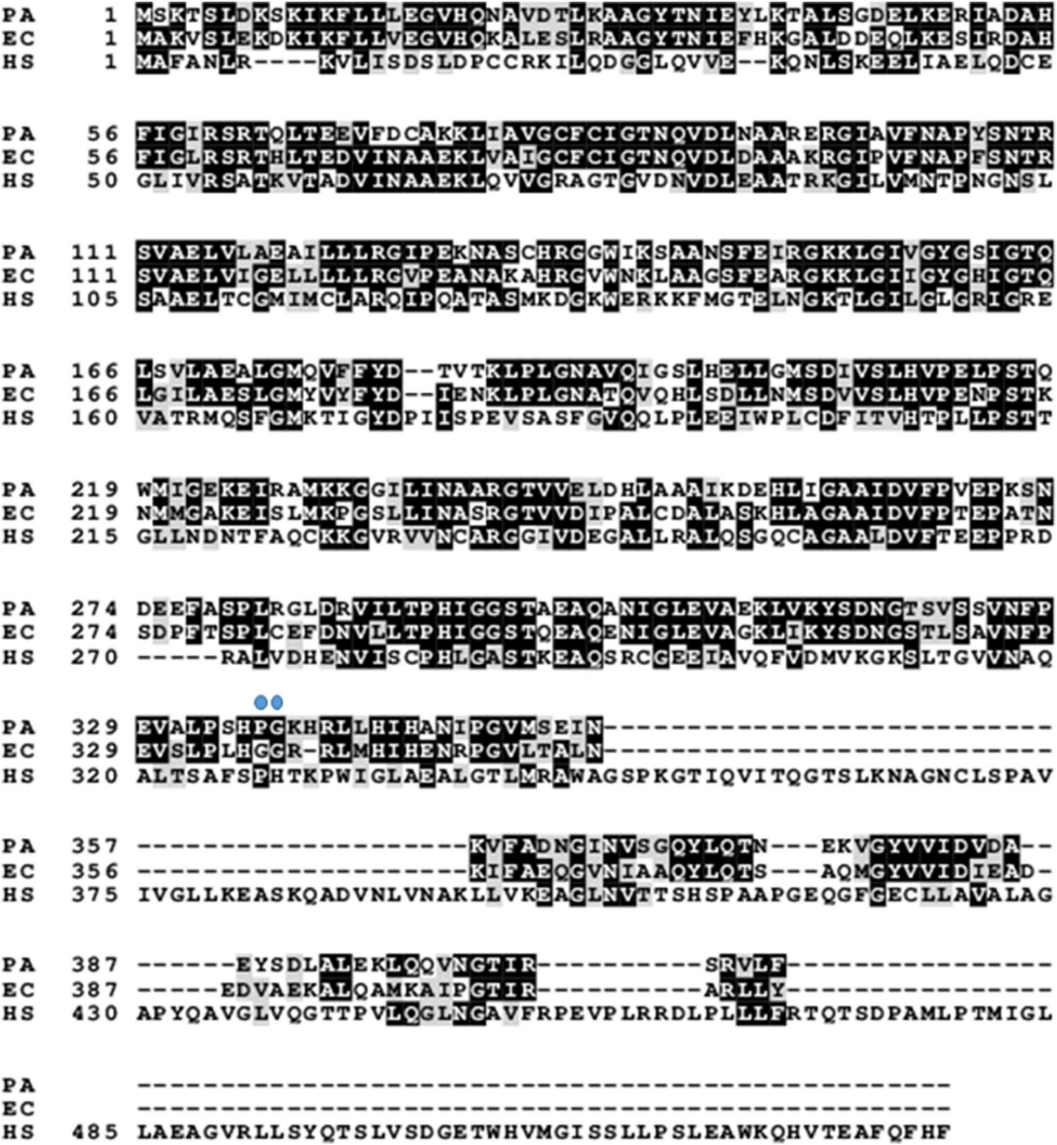
Sequence alignment of PGDH from *P. aeruginosa*, *E. coli*, and human. The number denotes the position of the amino acid residue in the sequence of *E. coli* PGDH from a previous report by Tobey and Grant [23]. The black boxes indicate identical residues, and the gray boxes show amino acid similarity. Blue dots show the Pro-336-Gly-337 at the connecting region between the ACT domain and the substrate-binding domain in *P. aeruginosa* PGDH [7, 8]. PA refers to *P. aeruginosa,* EC to *E. coli*, and HS to human

Figure 1 shows the amino acid sequence alignments of PGDHs from three species, *P. aeruginosa*, *E. coli*, and human. The similarity score is 64%, and the identity score is 81% between *P. aeruginosa* and *E. coli*. The similarity score is 24%, and the identity score is 37% between *P. aeruginosa* and human. The similarity score is 23%, and the identity score is 35% between *E. coli* and human. The human PGDH sequence is longer than the *P. aeruginosa* and *E. coli* PGDHs because of the presence of the insertion sequence (amino acids residues 348–394) in Fig. 1.

### SDS-PAGE analysis of the purified PGDHs

**Fig. 2:**
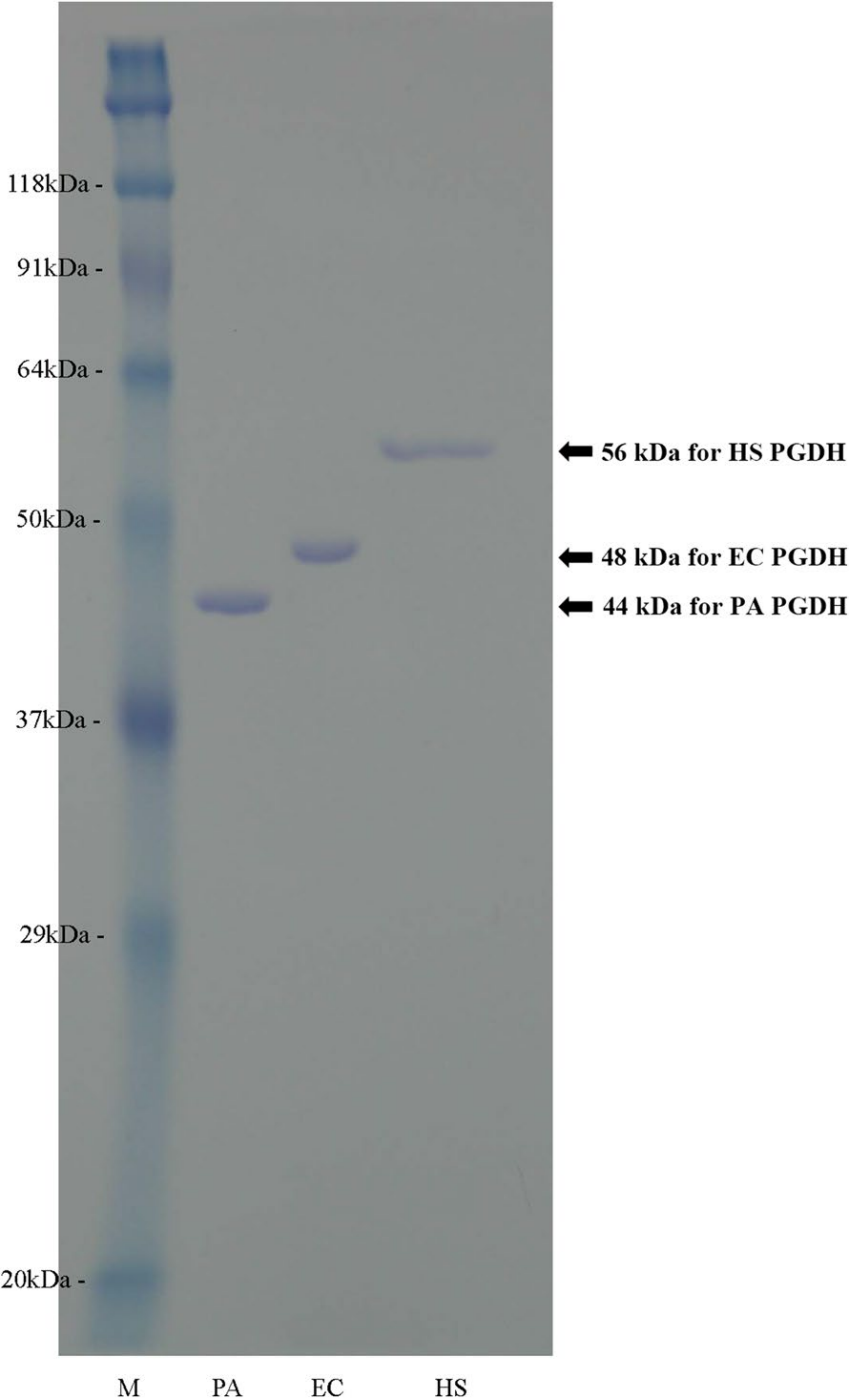
SDS-PAGE analysis for the purity of the recombinant PGDHs. The purified PGDHs from three species were separated on to a 12% SDS-PAGE followed by staining the gel with Rapid Stain CBB Kit (Nacalai Tesque). Lanes: M, molecular mass standards indicated on the left; PA, EC, and HS show the PGDHs from *P. aeruginosa, E. coli,* and human colon, respectively

The result of SDS-PAGE analysis of the three purified PGDHs is shown in Fig. 2. PGDHs of the three species were purified to the level of a single band without any visible contaminating band. The *P. aeruginosa* and the human PGDH bands appeared at the molecular weight size (44 kDa and 56 kDa) that was deduced from their *serA* gene sequences, while the *E. coli* PGDH band appeared at a slightly higher than the expected molecular weight size (48 kDa) predicted from its *serA* gene sequence.

### Optimum pH and ionic strength

**Fig. 3:**
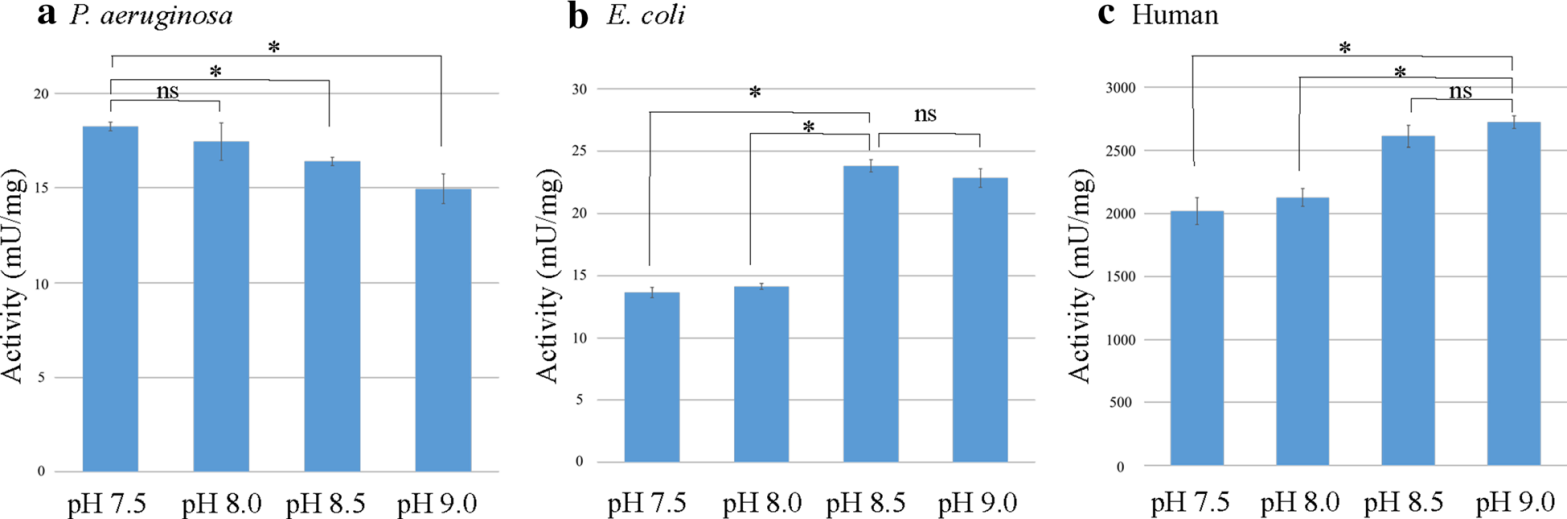
Optimum pH of the purified PGDHs derived from three different species. The optimal activity (mU/mg) of each PGDH was determined by performing the PGDH activity assay in the reaction mixture containing 100 mM Tris–HCl buffer at various pH. The assay was performed in triplicate, and data being shown as the mean activity (mU/mg) ± SD. **a** A significant difference in the activity of *P. aeruginosa* PGDH was observed between at pH 7.5 versus pH 8.5 and pH 9.0 (*P < 0.05), but not at pH 7.5 versus pH 8.0 (ns: not significant, P > 0.05). **b** A significant difference was observed in the activity of *E. coli* PGDH measured at pH 8.5 versus pH 7.5 and pH 8.0, (*P < 0.05), but not at pH 8.5 versus pH 9.0 (ns). **c** A significant difference was observed in the activity of human PGDH measured at pH 9.0 versus pH 7.5 and pH 8.0 (*P < 0.05), but not at pH 8.5 versus pH 9.0 (ns)

**Fig. 4:**
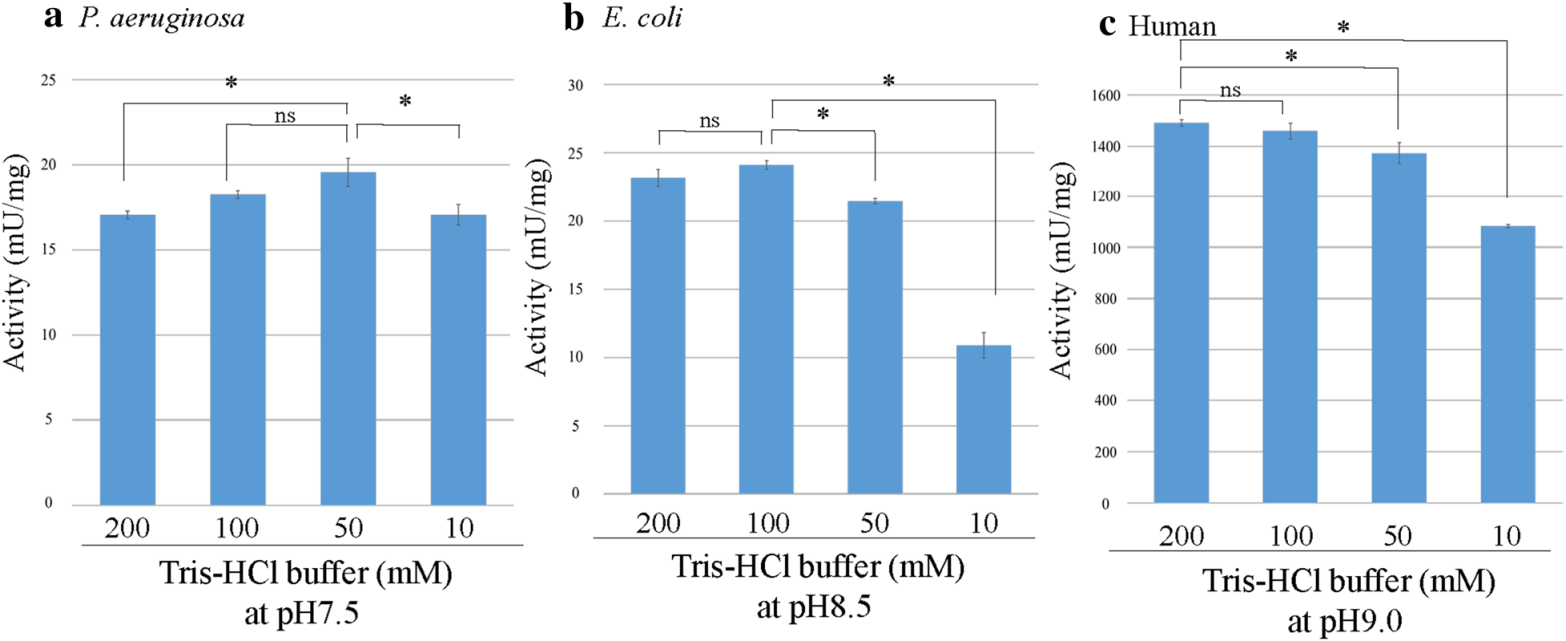
The optimum ionic strength of the purified PGDHs derived from three different species. The optimal activity (mU/mg) of each PGDH was determined by performing the PGDH activity assay in the reaction mixture containing various concentrations of Tris–HCl buffer. The assay was performed in triplicate, and data being shown as the mean activity (mU/mg) ± SD. **a** A significant difference was observed in the *P. aeruginosa* PGDH activity measured at 50 mM versus at 200 mM and 10 mM (*P < 0.05), but not between *P. aeruginosa* PGDH activity at 50 mM versus at 100 mM (ns: not significant, P > 0.05). **b** A significant difference was observed between *E. coli* PGDH activity at 100 mM and that at 50 mM and 10 mM (*P < 0.05), but not between *E. coli* PGDH activity at 100 mM and that at 200 mM (ns). **c** A significant difference was observed between human PGDH activity at 200 mM and that at 50 mM and 10 mM (*P < 0.05), but not between human PGDH activity at 200 mM and that at 100 mM (ns)

PGDHs activity shows different optimum pH and ionic strength depending on their origin species (Figs. 3, 4). As shown in Fig. 3, the optimal activity of *P. aeruginosa* PGDH was detected between pH 7.5 and pH 8.0, while that of both *E. coli* PGDH and human PGDH was between pH 8.5 and pH 9.0. Also, as shown in Fig. 4, the optimal activity of *P. aeruginosa* PGDH was observed in 50 and 100 mM Tris–HCl buffer, while that of both *E. coli* PGDH and human PGDH was detected in 100 and 200 mM Tris–HCl buffer.

### Inhibitory effect of l-serine on PGDH activity

**Fig. 5:**
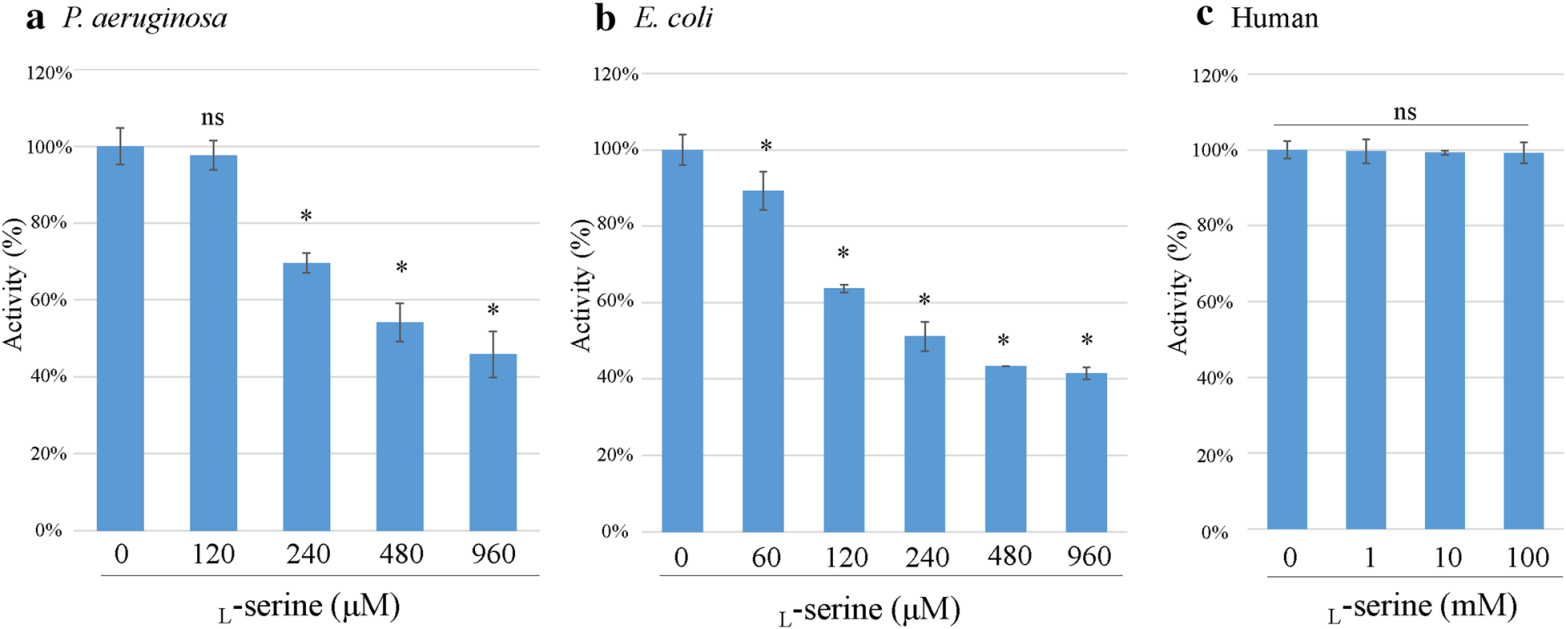
Inhibitory effect of l-serine on the PGDH activity. The optimal activity (mU/mg) of each PGDH was determined by performing the assay with different concentrations of l-serine. The assay was performed in triplicate. Data are shown as the ratio of the PGDH activity in the presence of l-serine to that without l-serine and is expressed as the mean % ± SD. **a** Significance difference in activity was observed between *P. aeruginosa* PGDH without serine and *P. aeruginosa* PGDH in the presence of 240 mM, 480 mM, and 960 mM serine (*P < 0.05), but not between *P. aeruginosa* PGDH without serine and *P. aeruginosa* PGDH in the presence of 120 mM serine (ns: not significant, P > 0.05). **b** A significant difference was observed between *E. coli* PGDH without serine and *E. coli* PGDH in the presence of 60 mM, 120 mM, 240 mM, 480 mM, and 960 mM serine (*P < 0.05). **c** A significant difference was not observed between human PGDH without serine and human PGDH in the presence of 1 mM, 10 mM, and 100 mM serine (ns)

**Fig. 6:**
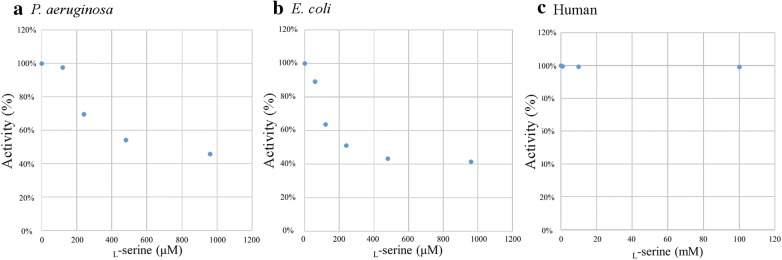
A graph plot shows for the PGDH activity (%) and the l-serine concentrations. The 50% inhibitory concentration (IC_50_) of l-serine was calculated from data shown in Fig. 5. The PGDH activity of *P. aeruginosa* (**a**), that of *E. coli* (**b**) and the human PGDH activity (**c**)

As shown in Fig. 5, the PGDH activity of *P. aeruginosa* was inhibited in a dose-dependent manner by 240–960 μM l-serine (P < 0.05) and that of *E. coli* was also inhibited in a dose-dependent manner by 60–960 µM l-serine (P < 0.05). As described previously [2], the 50% inhibitory concentration (IC_50_) of l-serine was calculated by plotting the inhibitor concentration against the percent activity of PGDH (Fig. 6). IC_50_ of l-serine against *P. aeruginosa* PGDH was 630 µM and that against *E. coli* PGDH was 250 µM. On the other hand, human PGDH activity was not inhibited even by the addition of 100 mM l-serine (Figs. 5, 6).

### Inhibitory effect of d-serine on PGDH activity

**Fig. 7:**
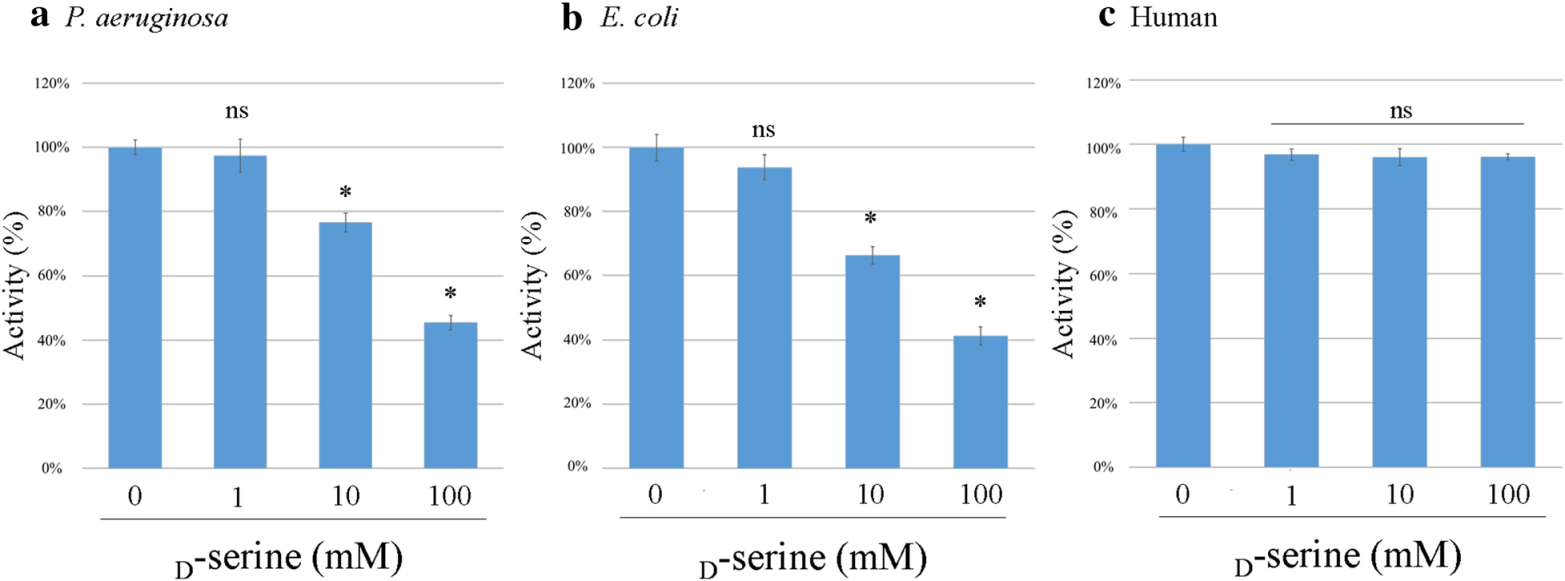
Inhibitory effect of d-serine on the PGDH activity. The optimal activity (mU/mg) of each PGDH was determined by performing the assay in the reaction mixture containing various concentrations of d-serine. The assay was performed in triplicate. Data are shown as the ratio of the PGDH activity in the presence of d-serine to that without d-serine and is expressed as the mean % ± SD. **a** Significance difference in activity was observed between *P. aeruginosa* PGDH without serine and *P. aeruginosa* PGDH in the presence of 10 mM and 100 mM serine (*P < 0.05), but not between *P. aeruginosa* PGDH without serine and *P. aeruginosa* PGDH in the presence of 1 mM serine (ns: not significant, P > 0.05). **b** A significant difference was observed between *E. coli* PGDH without serine and *E. coli* PGDH in the presence of 10 mM and 100 mM serine (*P < 0.05), but not between *E. coli* PGDH without serine and *E. coli* PGDH in the presence of 1 mM serine (ns, P > 0.05). **c** A significant difference was not observed between human PGDH without serine and human PGDH in the presence of 1 mM, 10 mM, and 100 mM serine (ns, P > 0.05)

**Fig. 8:**
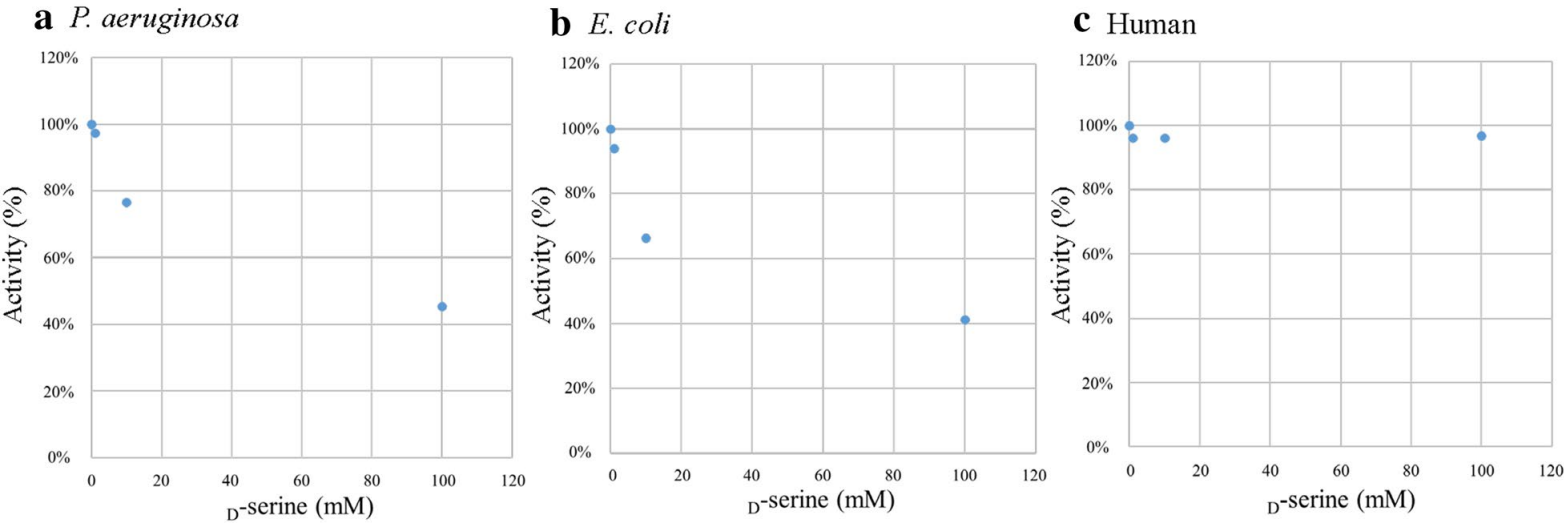
A graph plot shows for the PGDH activity (%) and the d-serine concentrations. The 50% inhibitory concentration (IC_50_) of d-serine was calculated from the data shown in Fig. 7. The PGDH activity of *P. aeruginosa* (**a**), that of *E. coli* (**b**) and the human PGDH activity (**c**)

As shown in Fig. 7, the PGDH activity of both *P. aeruginosa* and *E. coli* was inhibited in a dose-dependently manner by the addition of 10 to 100 mM d-serine (P < 0.05). As shown in Fig. 8, the IC_50_ of d-serine against *P. aeruginosa* PGDH was 76 mM and that against *E. coli* PGDH was 45 mM. On the other hand, the human PGDH activity was not inhibited even by the addition of 100 mM d-serine (Figs. 7, 8).

## Discussion

In the present study, the optimal activity of *P. aeruginosa* PGDH was detected at between pH 7.5 and pH 8.0, with a salt concentration range between 50 and 100 mM Tris–HCl buffer. Since the optimum conditions for the *P. aeruginosa* PGDH activity have not been reported yet, our results would be a useful reference for the future study. Also, optimum pH 8.5 for the *E. coli* PGDH determined in the present study is consistent with the previous report [6]. On the other hand, the optimum pH for the human colon PGDH has not been reported yet, but the activity assay of human PGDH was carried out at pH 7.1 [16, 17]. In the present study, the optimum pH for the human PGDH was 8.5–9.0 and is different from the pH 7.1 used in the previous reports. There seems to be no difference in the sequence of PGDH derived from human colon cDNA in the present study and that derived from HeLa cells and skin-derived fibroblasts reported in earlier studies including a recent review on PGDH [8, 16, 17] (GenBank reference sequence NM_006623.3 [16] and AF006043.1 [17]). Taken together, it is suggested that the difference in optimum pH for the human PGDH might be due to the differences in the purification and PGDH assays rather than a difference in the tissue type from which the PGDH was isolated. In earlier studies [16, 17], the human PGDH activity was measured by using crude extracts prepared from transiently transfected HEK293T or BHK-21 cells expressing human PGDH and by following the previously reported methods [18, 19], where the reaction mixture contained 25 mM HEPES, pH7.1, in the PGDH activity assay. On the other hand, we evaluated the human PGDH activity by following the previously reported methods to determine the bacterial PGDH activity and its inhibition by l-serine [13]. Therefore, we need to investigate further for the optimum pH for the human PGDH by following the previously reported methods [18, 19] in the future study.

As described in detail previously [7, 8], on the basis of the results of amino acid sequence alignments shown in Fig. 1, the basal structure of *P. aeruginosa* PGDH belongs to the type II motif, as well as that of *E. coli* PGDH. On the other hand, human PGDH is classified as the type I motif, with the extended C-terminal region [7, 8]. As for inhibition by l-serine is concerned, the PGDH activity of both *P. aeruginosa* and *E. coli* was inhibited in a dose-dependent manner by the addition of l-serine, but the human PGDH activity was not inhibited even at 100 mM l-serine, which further corroborated the findings of the previous studies  [7, 8]. It is hypothesized that this difference might be due to the difference in the length of the regulatory domain because in type I motif, there is an insertional ASB domain, which is not present in type II motif [7, 8]. Further studies are needed to confirm this by utilizing a chimera type II PGDH construct from *P. aeruginosa* in which the regulatory domain is substituted with that of type I motif and investigate whether the PGDH activity of the chimera type II PGDH from *P. aeruginosa *is affected by the addition of 100 mM l-serine.

On comparison of the IC_50_ values of l-serine against *P. aeruginosa* and *E. coli* PGDH, the *E. coli* PGDH showed higher sensitivity to inhibition by l-serine. As described in detail previously [20], mutations of Gly-336–Gly-337, at the connecting region between the ACT domain and the substrate-binding domain, significantly reduced the inhibitory effect of l-serine on the *E. coli* PGDH activity. On the other hand, in *P. aeruginosa* PGDH, a Pro-Gly sequence was found corresponding to Gly-336–Gly-337 of *E. coli* PGDH at the connecting region between the ACT domain and the substrate-binding domain as shown in Fig. 1. Taken together, it is predicted that the difference in IC_50_ values of l-serine against *P. aeruginosa* PGDH and *E. coli* PGDH might be due to the presence of the Gly–Gly sequence in *E. coli* PGDH. However, further experiments are required to confirm this hypothesis by characterizing a mutant PGDH from *P. aeruginosa* that has a Gly–Gly sequence and examining the inhibitory effect of l-serine on the mutant PGDH.

In the present study, we investigated the inhibitory effect of d-serine, which is an amino acid enantiomer of l-serine, against the PGDH activity. As a result, the IC_50_ of d-serine was much higher than that of l-serine; IC_50_ of d-serine against PGDHs of *P. aeruginosa* and *E. coli* were 120- and 180-fold higher than that of l-serine against PGDHs of *P. aeruginosa* and *E. coli*, respectively. As described in detail previously [21, 22] earlier the inhibitory effect of other amino acids including l-serine, glycine, l-alanine, l-cystine, and l-homoserine on the *E. coli* PGDH activity was evaluated, and it was found that l-serine had the lowest IC_50_ value among other amino acids .

## Conclusions

We previously reported that the inhibition of the *serA* gene by l-serine caused significant reduction in the bacterial penetration through the Caco-2 cell monolayers, bacterial swarming and swimming motilities, bacterial adherence to Caco-2 cells, and virulence in flies in the wild-type *P. aeruginosa* PAO1 strain. Oral administration of l-serine to the compromised hosts, through the inhibition of *serA* function, might have the potential to prevent the bacterial infection and septicemia caused by *P. aeruginosa* [1]. Here, we showed that l-serine inhibits the activity of PGDH from *P. aeruginosa* PAO1 strain, while l-serine did not affect the activity of human PGDH even at 159-fold higher concentration (100 mM) when compared with IC_50_ value of l-serine against *P. aeruginosa* PGDH (630 µM) (Figs. 5, 6). Overall, our data suggest that the oral administration of l-serine to the compromised human hosts might have the potential to interfere with the bacterial translocation and prevent the septicemia caused by *P. aeruginosa* through the inhibition of PGDH activity of SerA protein.

## Data Availability

All data generated for this study are included in the published article.
